# The relevance of pedigrees in the conservation genomics era

**DOI:** 10.1111/mec.16192

**Published:** 2021-10-22

**Authors:** Stephanie J. Galla, Liz Brown, Yvette Couch‐Lewis (Ngāi Tahu: Te Hapū o Ngāti Wheke, Ngāti Waewae), Ilina Cubrinovska, Daryl Eason, Rebecca M. Gooley, Jill A. Hamilton, Julie A. Heath, Samantha S. Hauser, Emily K. Latch, Marjorie D. Matocq, Anne Richardson, Jana R. Wold, Carolyn J. Hogg, Anna W. Santure, Tammy E. Steeves

**Affiliations:** ^1^ Department of Biological Sciences Boise State University Boise Idaho USA; ^2^ School of Biological Sciences University of Canterbury Christchurch Canterbury New Zealand; ^3^ New Zealand Department of Conservation Twizel Canterbury New Zealand; ^4^ Te Rūnanga o Ngāi Tahu Te Whare o Te Waipounamu Christchurch Canterbury New Zealand; ^5^ New Zealand Department of Conservation Invercargill Southland New Zealand; ^6^ Smithsonian‐Mason School of Conservation Front Royal Maryland USA; ^7^ Center for Species Survival Smithsonian Conservation Biology Institute National Zoological Park Washington District of Columbia USA; ^8^ Department of Biological Sciences North Dakota State University Fargo North Dakota USA; ^9^ Department of Biological Sciences University of Wisconsin‐Milwaukee Milwaukee Wisconsin USA; ^10^ Department of Natural Resources and Environmental Science Program in Ecology, Evolution and Conservation Biology University of Nevada Reno Reno Nevada USA; ^11^ The Isaac Conservation and Wildlife Trust Christchurch Canterbury New Zealand; ^12^ School of Life and Environmental Sciences University of Sydney Sydney NSW Australia; ^13^ School of Biological Sciences University of Auckland Auckland Auckland New Zealand

**Keywords:** conservation genomics, ex situ, in situ, kinship, pedigree, quantitative genetics

## Abstract

Over the past 50 years conservation genetics has developed a substantive toolbox to inform species management. One of the most long‐standing tools available to manage genetics—the pedigree—has been widely used to characterize diversity and maximize evolutionary potential in threatened populations. Now, with the ability to use high throughput sequencing to estimate relatedness, inbreeding, and genome‐wide functional diversity, some have asked whether it is warranted for conservation biologists to continue collecting and collating pedigrees for species management. In this perspective, we argue that pedigrees remain a relevant tool, and when combined with genomic data, create an invaluable resource for conservation genomic management. Genomic data can address pedigree pitfalls (e.g., founder relatedness, missing data, uncertainty), and in return robust pedigrees allow for more nuanced research design, including well‐informed sampling strategies and quantitative analyses (e.g., heritability, linkage) to better inform genomic inquiry. We further contend that building and maintaining pedigrees provides an opportunity to strengthen trusted relationships among conservation researchers, practitioners, Indigenous Peoples, and Local Communities.

## 1 INTRODUCTION

### 1.1 The conservation genetics toolbox

Since the late 1960’s, conservation genetics has grown from a handful of techniques into a fully‐fledged discipline that uses genetic information to inform the conservation management of threatened species worldwide (Avise, 2008). This field has developed a substantive toolbox applied to understand phylogenetics and species delimitation (Coimbra et al., 2021; Yusefi et al., 2020), population structure and demographics (Coimbra et al., 2020), natural community profiling (Young et al., 2020), and the level of standing genetic variation within and among populations (Zhang et al., 2020). Much discussion regarding the conservation genetic toolbox has been dedicated to the types of variants that are used for genetic inference, and for good reason: in a relatively short time frame, the field has experienced remarkable growth, from detecting variants using allozyme protein electrophoresis to detecting hundreds of thousands of variants through high throughput sequencing (HTS) approaches (Hohenlohe et al., 2021). While the field of conservation genetics was founded on managing putatively neutral diversity as a proxy for evolutionary potential (Yoder et al., 2018), new HTS sequencing and computational tools make it possible for researchers to elucidate the genomic basis of functional traits important to adaptation, which has implications to understanding how species may respond to a changing world (Hoelzel et al., 2019; Mable, 2019).

As HTS continues to advance, there will no doubt be more new and exciting tools incorporated into conservation genetic inquiry. In addition to new methods enabled by advances in HTS, there remains one long‐standing tool within the conservation genetics toolbox that is often overlooked: the pedigree. Pedigrees, or documented ancestry of individuals in a population, have been recorded by humans for millennia. These family trees have long provided a proxy for understanding the transmission of traits from generation to generation and maintain diverse applications in agriculture (e.g., Smith et al., 2004), human health (Bennett, 2011), evolutionary biology (Kruuk & Hill, 2008), and conservation (e.g., Ballou et al., 2010). Sewall Wright famously advanced the utility of pedigrees through his contributions towards pedigree‐based path analysis, inbreeding, and kinship estimates (Ballou, 1983; Wright, 1922). Assuming Mendelian inheritance, pedigrees provide an estimate of kinship as the probability of alleles being identical‐by‐descent (IBD) from a common ancestor (Lacy, 1995). As the fields of conservation biology and genetics emerged in the second half of the 20th century, these same principles were used to estimate kinship, inbreeding, and heritability of functional traits in threatened populations in an effort to conserve evolutionary potential.

### 1.2 Pedigrees in conservation management

**FIGURE 1:**
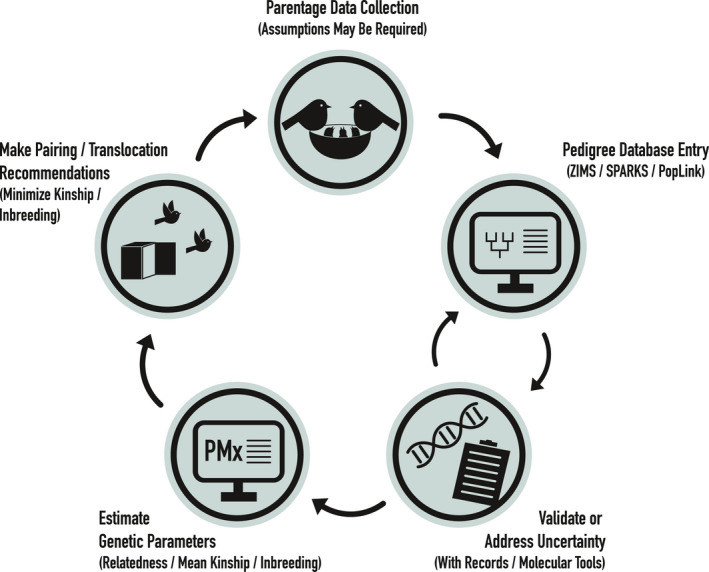
Schematic detailing one use of pedigrees in conservation breeding programs to maximize genome‐wide diversity. Parentage data is collected in ex situ or in situ environments, entered into databases using readily available software, validated when necessary using records and molecular tools, used to estimate genetic parameters, and inform conservation decisions

Pedigrees have been particularly well suited for the genetic management of captive (ex situ) or intensively managed wild (in situ) or semi‐wild (“sorta” situ; Wildt et al., 2019) populations of animals and plants, where ancestry is more reliably documented. Pedigree use is exemplified by the zoo and aquarium community, who have built a data‐driven paradigm of pedigree‐based management, including user‐friendly software to manage pedigree information (e.g., SPARKS, PopLink, ZIMS; Faust et al., 2019, Species360 2021) and to calculate pedigree‐based genetic statistics (e.g., PMx, Lacy et al., 2012). Given these readily available tools, and the relative ease and low cost of maintaining parentage information for many species being intensively managed, pedigrees offer an achievable means of managing genetic diversity in these populations. Pedigree‐based conservation management is often kinship‐based, with the coefficient of kinship (*f*) being a metric of the coefficient of relatedness (*R*, *R* = 2*f* in the absence of inbreeding; Lacy, 1995, 2009). Common pedigree‐based statistics for small population management include mean kinship (i.e., MK, or the average kinship of one individual to all others in a population, including itself), inbreeding coefficients (*F*), founder genome‐equivalents (i.e., the effective number of individuals founding a population), and population‐level gene diversity (GD, also known as expected heterozygosity; Ballou et al., 2010; Lacy, 1995). These statistics are frequently used to track loss of founder alleles over time (e.g., MacCluer et al., 1986) and minimize mean kinship and inbreeding in threatened populations to maintain the evolutionary potential of the species of interest (Galla et al., 2020; Ivy et al., 2009; Willougby et al., 2015; Figure 1). Indeed, studies have shown the efficacy of this approach (Lacy, 2009), with pedigrees being used to measure and manage diversity and inbreeding in animals worldwide, including Atlantic and sockeye salmon (*Salmo salar* and *Oncorhynchus nerka*, respectively; O’Reilly & Kozfkay, 2014), Tasmanian devil (*Sarcophilus harrisii*; McLennan et al., 2018; Wright et al., 2021), American bison (*Bison bison*; Giglio et al., 2018), whooping crane (*Grus americana*; Boardman et al., 2017), takahē (*Porphyrio hochstetteri*; Grueber & Jamieson, 2008), and Houbara bustard (*Chlamydotis undulata undulata*; Rabier et al., 2020). When pedigrees are complete and accurate, they have been shown to explain more variation in inbreeding than microsatellites do (Nietlisbach et al., 2017) and provide similar estimates of relatedness to thousands to tens of thousands of genome‐wide single nucleotide polymorphisms (i.e., SNPs; Galla et al., 2020). While pedigrees have been extensively used to manage genome‐wide diversity of animals in zoos and aquaria, a recent review by Wood et al. (2020) has highlighted their potential for managing diversity and viability for plant collections and seed banks. Ongoing efforts are being made to optimize collections and maintain plant material ex situ for long‐term conservation and potential use in future restoration (Di Santo & Hamilton, 2020). The goals of these collections are to both preserve diversity representative of in situ population differences across a species’ range, and to ensure that ex situ population genetic variation is maintained to preserve adaptive evolutionary potential (Di Santo & Hamilton, 2020; Hamilton et al., 2020). Given the overlapping goals —but differing approaches— of plant and animal conservation breeding programs, we anticipate that zoo, aquaria, and botanical communities will learn much from one another as different approaches are developed and tested.

Most zoos and aquaria use pedigrees in a well‐supported paradigm of measuring and managing putatively neutral genome‐wide diversity, but pedigrees have also been used to characterize and manage functional diversity within ex situ plant and fisheries systems. For example, in 1983 the American Chestnut Foundation embarked on an ambitious breeding program to backcross blight‐susceptible American chestnut (*Castanea dentata*) —a species on the brink of extinction— with blight‐resistant Chinese chestnut (*C*. *mollisima*). In this instance, ancestry data from pedigrees and phenotypic data on blight resistance were used for crossing programs, based on the hypothesis that blight resistance had a genetic basis (Westbrook et al., 2020). In addition to disease resistance, pedigrees are often used in greenhouses or intensively managed common gardens to understand the functional ability of plants to cope with stress through gene‐by‐environment (i.e., GxE) experiments (e.g., George et al., 2020). These experiments aim to disentangle genetic (as derived from pedigree‐based kinship) and environmental contributions, and their interactions, to explain phenotypes of interest in individuals. Conservation biologists can then use predictions of local environmental conditions in the short‐ to medium‐term to select well‐adapted individuals or varieties for conservation translocations and restoration (e.g., Richardson & Chaney, 2018).

Pedigree data has also advanced our understanding of wild populations and ability to manage them (i.e., in situ or sorta situ conservation; Kruuk & Hill, 2008; Wildt et al., 2019). For a pedigreed natural population of Florida scrub jays (*Aphelocoma coerulescens*), researchers used pedigrees to predict the effects of selection and gene flow on how declining populations might evolve in a short time period (Chen et al., 2019). A wild pedigree for grey wolves (*Canus lupus*) in Yellowstone National Park combined with phenotypes recorded for those individuals led to advances in understanding the heritability of behaviour and the genetic basis of mange in this iconic species (DeCandia et al., 2021; vonHoldt et al., 2020). Pedigrees have also been used in Eastern Massasauga rattlesnakes (*Sistrurus catenatus*) to elucidate dispersal and connectivity between populations, with implications for restoration and translocation efforts (Martin et al., 2021). Important to conservation efforts for small and isolated wild populations is the ability to re‐establish gene flow using conservation translocations (i.e., genetic rescue; Ralls et al., 2020). Documented ancestry of wild populations can help refine estimates of effective population size, social group structure, genetic connectivity among populations, and potential local adaptation amongst populations, which can aid in designing successful translocation programs that minimize inbreeding depression while avoiding outbreeding depression. For example, pedigrees have been used to inform genetic conservation or rescue efforts for wild populations of black‐tailed prairie dogs (*Cynomys ludovicianus*; Shier, 2006), Rocky Mountain bighorn sheep (*Ovis canadensis*; Hogg et al., 2006), Scandanavian grey wolves (Åkesson et al., 2016), and Tasmanian devils (McLennan et al., 2020). From these examples, pedigrees have continued to provide an invaluable resource for understanding and managing diversity in plants and animals. However, there are pitfalls for pedigrees that can affect their accuracy and utility for conservation efforts.

### 1.3 Pedigree pitfalls and solutions

One common assumption of pedigrees is that the founding individuals (hereafter, founders) are equally unrelated. As many threatened populations have experienced significant declines before populations are pedigreed, founders from these populations may include more variance in kinship amongst one another than individuals randomly sampled from nonthreatened populations. For instance, a study of microsatellite‐based relatedness in kākāpō (*Strigops habroptilus*) demonstrated that full‐sibling and half‐sibling relationships were represented amongst founders (Bergner et al., 2014), which has been confirmed with thousands of genome‐wide SNPs (New Zealand Department of Conservation, unpublished data). Research in Tasmanian devil has further shown that inferring estimates of relatedness amongst founders using molecular and geographic data revealed significantly higher inbreeding coefficients in the years following establishment, compared to pedigrees that treat founders as equally unrelated (Hogg et al., 2019). While these effects are ameliorated with higher pedigree depth from founding individuals (Balloux et al., 2004), this assumption can be perpetuated when individuals of unknown ancestry (e.g., supplemented individuals, or individuals with missing information) are incorporated into the pedigree in later generations. In addition to these issues, pedigrees are also susceptible to human transcription errors, which can compound in pedigrees over time (Hammerly et al., 2016).

Missing information is often a concern for wild pedigrees where parentage is difficult to ascertain. For example, accurate parentage can be challenging due to the time and cost required to monitor breeding individuals in the wild, coupled with the dispersal potential and breeding behaviour of the organism (e.g., polygamous species like flock breeding birds, herd‐breeding mammals, and open‐pollinated plants; Ashley, 2010; Ivy et al., 2016; Wildt et al., 2019). This challenge is exacerbated when individuals lose their identifiers (e.g., radio collars, tags, or leg bands; Milligan et al., 2003) or when socially monogamous individuals participate in extra‐pair parentage (e.g., Overbeek et al., 2020). Even in captivity, parentage amongst polygynous breeders is difficult to track. Individuals with unknown parentage are either excluded from the potential breeding population due to kinship uncertainty, are assigned a multiple parentage average kinship value (i.e., MULTs; Lacy, 2012), or are assigned with assumed parents (Ballou et al., 2010). Building MULTs into a pedigree has been shown to retain greater genetic diversity while still maintaining inbreeding avoidance as opposed to removing individuals of unknown parentage, as shown in Arabian oryx (*Oryx leucoryx*; Putnam & Ivy, 2014). However, for species that experience large differences in reproductive success between individuals, the MULT approach has been shown to both over‐ and underestimate individual genetic contributions (e.g., Tasmanian devil; Farquharson et al., 2019). To highlight the severity of such challenges in group breeding species, over 50% of ungulate species managed under the auspices of the Association of Zoos and Aquariums Species Survival Plan (SSP) have less than half of their pedigree known; in fact, only 15% have completely known pedigrees (R. M. Gooley and E. K. Latch, unpublished data). This is a common challenge encountered with group‐housed captive populations, which can lead to management challenges with respect to kinship calculations, breeding recommendations, and genetic diversity retention (Hauser et al., 2021). Resolving unknown parentage and generating accurate kinship values would lead to more effective conservation management of intensively managed wild and captive populations.

To address pedigree challenges, molecular (e.g., genetic or genomic) estimates of relatedness from can be used to complete and complement pedigrees. For relatively diverse wild populations, microsatellite‐based approaches can be informative (McLennan et al., 2018), especially for inferring close relatives. For example, a recent study from Moran et al., (2021) highlighted the benefit of using microsatellite markers to verify parentage in critically endangered California condor (*Gymnogyps californianus*). While microsatellites are useful, high density single nucleotide polymorphisms (i.e., SNPs) generated through high throughput sequencing approaches (i.e., HTS) often provide better resolution for estimating identity‐by‐descent, even when populations are inbred or relationships are more distant (Allendorf et al., 2010; Flanagan & Jones, 2019; Galla et al., 2020; Taylor, 2015). Molecular data have been used to enhance pedigrees for use in conservation management, including estimating founder relationships (Hogg et al., 2019) and reconstructing pedigrees (Gooley et al., 2017; Huisman, 2017; McLennan et al., 2018) in Tasmanian devils and guiding metapopulation management of pedigree and nonpedigree populations of sable antelope (*Hippotragus niger*) and dama gazelle (*Nanger dama*; R. M. Gooley, personal communication). Molecular data can also be used to validate uncertain and semi‐wild pedigrees. For example, microsatellite markers have recently been used to validate pedigrees for intensively‐monitored and critically endangered wild populations of kakī/black stilt (*Himantopus novaezelandiae*; Overbeek et al., 2020) and black robin (*Petroica traversi*; Forsdick et al., 2021). Even when pedigrees are ‘perfect’ with no missing information and accurate parentage assignments, genomic data may capture more existing variation in realized relatedness between relatives, given the effects of recombination and segregation and random fertilisation (Speed & Balding, 2015). However, the extent to which these differences impact conservation management decisions including pairing recommendations remains to be explored (Galla et al., 2020).

**FIGURE 2:**
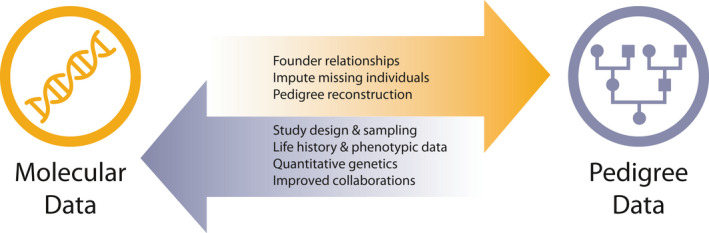
Diagram illustrating the mutual benefits between molecular (e.g., genetic/genomic) and pedigree data

Fellow conservation practitioners and researchers have queried whether time and resources should be dedicated towards building pedigrees from scratch and maintaining them, considering their caveats and the abundance of HTS data available to produce estimates of relatedness that are comparable to pedigrees (Speed & Balding, 2015). In this perspective, we argue that pedigrees remain an invaluable tool in the conservation genomics era, providing an affordable approach to estimate relatedness and inbreeding (Nietlisbach et al., 2017, but see also Kardos et al., 2015), enhance genomic inquiry, and capture important metadata that genomic data alone cannot (Figure 2). Based on our collective experience, we assert that gathering the behavioural and ecological data that underlie pedigrees not only advances our understanding of interindividual relationships, but also develops strong ties between conservation geneticists, practitioners, and beyond (see below). In the conservation genomics era, we posit that the greatest conservation genomic advances will happen when we invest in both pedigree and genomic data.

## 2 THE VALUE OF PEDIGREES IN THE CONSERVATION GENOMIC ERA

### 2.1 Beyond kinship: Demographic information and metadata captured in pedigrees

Pedigrees capture a wealth of information beyond individual relationships produced purely by genomic data (Clutton‐Brock & Sheldon, 2010). The behavioural and ecological observations required to provide interindividual relationships results in a rich ancillary data set that cannot be captured by genomic data alone. For example, pedigrees can discern different relationships with identical relatedness coefficients (e.g., half‐siblings compared to grandparent‐grandoffspring, *R* = 0.25), which can have different social and ecological consequences. Pedigrees also carry rich demographic data that may be inaccessible from molecular data, including sex for species without genetic sex determination (Janzen & Paukstis, 1991), cohort, number of offspring, age, and survival. Information regarding number of offspring produced throughout a pedigree is valuable for understanding individual metrics of fitness and genetic contributions, as shown in Florida scrub jays (Chen et al., 2019), song sparrows (*Melospiza melodia*; Reid et al., 2019), and Soay sheep (*Ovis aries*; Hunter et al., 2019). In addition to demographic information, phenotypic data collected alongside ancestry is often extensive, including morphometrics (e.g., weight, size, body condition), cause of death, behaviour, and signs of inbreeding depression (e.g., disease susceptibility, infertility). On its own, the metadata captured alongside pedigrees can be used to forecast best management practices for small populations through population viability analysis (i.e., PVA; Lacy & Pollak, 2021) and provides a critical resource for understanding demography and fitness (e.g., variance in reproductive success). We acknowledge that, in the absence of pedigree data, demographic data can be optionally collected independent of pedigrees along with genomic data for downstream analyses. However, these data sets rarely encompass the long‐term sampling inherent in pedigree records, and may not be able to accurately capture key evolutionary parameters including individual fitness. In contrast, and by virtue of how pedigree data is collected and structured, the long‐term demographic data associated with pedigrees is invaluable. For example, a recent study harnessed pedigree data from 15 species (>30 K individuals) to show how generations in captivity impact survival (Farquharson et al., 2021). Another study assessing breeding in 39 pedigreed populations of 21 wild animal species (>35 K females) concluded that many species were able to buffer annual fluctuations in optimal breeding date through phenotypic plasticity (de Villemereuil et al., 2020). Meta‐analyses on this scale would be impossible to ascertain using genomic data alone, given these studies rely on life history data carried in pedigrees. Further, metadata readily captured in pedigrees can also be integrated with genomic approaches, for example the construction of linkage maps and quantitative trait locus mapping (Pelgas et al., 2011; Sewell et al., 1999), genome‐wide association studies (GWAS; Morris et al., 2013), assessing adaptive potential (de Villemereuil, Rutschmann, Lee, et al., 2019), genomic selection (GS) studies, and GxE studies to test genotypes for association with environmental variation (Crossa et al., 2017; see below).

### 2.2 Pedigrees complement genomic study design and inference

The relationship information and metadata captured by pedigrees are an invaluable tool to help design and implement genomic research. For example, pedigrees provide biologically‐relevant data to inform a nonbiased selection of individuals for building representative and high‐quality reference genomes. When selecting an individual for a reference genome for species with genetic sex determination, some researchers have preferred selecting either the homogametic sex to ensure adequate coverage of the homogametic sex chromosome (i.e., X or Z), or the heterogametic sex to capture the alternative and often highly repetitive sex chromosome (i.e., Y or W; Rhie et al., 2021; Tomaszkiewicz et al., 2017). In addition to helping select the candidates for sampling based on sex, pedigrees can also identify individuals that are likely to be highly inbred, which assists with genome assembly by reducing error associated with ambiguity between heterozygosity and genetic paralogues (Hahn et al., 2014; Rhie et al., 2021). Further, detailed pedigrees can enable selection of parent‐offspring trios to generate phased de novo genome assemblies (Korbel & Lee, 2013; Koren et al., 2018; Leitwein et al., 2020). High‐quality de novo reference genomes are a powerful resource for the conservation and evolutionary genomics community by facilitating read mapping (Card et al., 2014), mining for genes of interest (e.g., Greenhalgh et al., 2021), and SNP discovery and genotyping (e.g., Brandies et al., 2019; Galla et al., 2019; Gooley et al., 2020). To further characterise variants across the genome, including structural variants (SVs), pedigrees may be used to inform the curation of a pangenome, which is the assembly of multiple individuals with the aim to capture all standing genomic diversity in a population or species of interest (Brockhurst et al., 2019; Tettelin et al., 2005). In this instance, a pedigree can be leveraged to identify distantly related individuals to ensure the pangenome is representative (Wold et al., 2021). Finally, pedigree‐based linkage maps have been a valuable resource for scaffolded genome assemblies by enabling higher assembly accuracy, order, and contiguity (Catchen et al., 2020).

Pedigree data is also an invaluable resource for selecting individuals for resequencing (i.e., whole genome resequencing, or WGS). For example, a pedigree can inform the choice of closely related family groups for genomic inquiry (e.g., Galla et al., 2020), understanding characterized phenotypes of interest (Nersisyan et al., 2019), or when maximizing representative genomic diversity across a species (Robinson et al., 2021). In the case of sable antelope (*Hippotragus niger*; Gooley et al., 2020) the software program PedSam (https://sites.uwm.edu/latch/software‐2/) was used to streamline the selection of individuals representative of founder diversity across many managed populations for downstream diversity comparisons. In a recent study in California condors, individuals with low inbreeding and kinship coefficients were selected using the pedigree, and were compared in terms of runs of homozygosity using WGS (Robinson et al., 2021). When familial relationships are known via pedigrees, this information can also be used to validate whether molecular approaches (e.g., extraction, amplification, library preparation, or sequencing) produce data that are consistent with biologically‐relevant expectations or experienced error along the way (see Galla et al., 2020 for details).

Beyond informing the individuals sampled for molecular studies, pedigrees can be pivotal to successful genetic variant discovery. For many conservation genomic research projects, variants (e.g., SNPs, SVs) are used as markers to identify and measure diversity (Hohenlohe et al., 2021; Wold et al., 2021). Artefacts from library preparation, sequencing, and bioinformatic processing can lead to false variants in data sets, which can bias downstream analyses (O'Leary et al., 2018). In addition to adequate filtering for sequencing depth and Hardy‐Weinberg equilibrium, validated pedigrees can be used as one tool for filtering false data sets from variants using Mendelian inheritance. This approach has long been used in the field of human genetics for marker validation, and in one study, was able to reduce marker error rates by 50% (Chen et al., 2013). A study in the pedigreed population of Florida scrub jays shows great promise for this approach, identifying sex‐linked and false SNPs from a reduced representation data set (Chen et al., 2014). Further, variant discovery for the critically endangered kākāpō is being informed by Mendelian inheritance, creating a high quality variant data set for all individuals of this species (J. Guhlin, personal communication). Because genomic research for species of conservation concern is often budget‐constrained, data sets are often hampered by low sequencing depth and subsequent missing data. In the fields of human and crop genetics, imputation (e.g., completing missing data sets with likely alleles using algorithms) is one option for addressing large amounts of missing data (Hickey et al., 2012; Sargolzaei et al., 2014). When coupled with genotypic information from family groups, this approach can increase the likelihood of accurate imputation, even of rare alleles (Ullah et al., 2019). We anticipate imputation—despite its caveats (see Roshyara et al., 2014)—will be explored more in the field of conservation genomics, especially for species with large genomes that are costly to sequence at high depths (e.g., some fish, insects, and plants; Mao et al., 2020) or as a cost‐effective option for conservation programs that can only sequence at low depths.

### 2.3 Pedigrees and quantitative genetics

Even in the absence of molecular information, pedigrees have long provided an understanding of the genetic basis of phenotypic differences between individuals in a population, the selective pressures on these traits, and evolutionary potential (e.g. Farquharson et al., 2017). Quantitative genetic models leverage variation in pedigree‐based relatedness between individuals to estimate the proportion of trait variation that is due to genetic differences between individuals (i.e., heritability; often estimated from complex pedigrees using a mixed effects statistical model termed the “animal model”, Wilson et al., 2010). In general, high heritabilities enable populations to evolve more quickly in response to selection, which is a valuable characteristic for threatened populations experiencing strong selection pressures due to rapid global change (Woodruff, 2001). For example, a recent study in hihi (*Notiomystis cincta*) used pedigree‐based heritability to reveal low evolutionary potential in this threatened bird (de Villemereuil, Rutschmann, Lee, et al., 2019). In addition to heritability, known family groups or distinct populations have also been essential to understanding GxE interactions, especially in plant species (Bisbing et al., 2020; Yoko et al., 2020). Empirical estimates of relatedness can be used instead of pedigrees, and may overcome pedigree pitfalls while providing realized estimates of genome‐sharing (Hill & Weir, 2011, 2012; Speed & Balding, 2015). However, genomic approaches often fail to account for important confounding effects which pedigrees naturally capture, including maternal and cohort effects (e.g., de Villemereuil et al., 2019) that may lead to biased estimates of heritability and evolutionary potential. Further, the one‐off design of many genomic studies, compared to the longer‐term monitoring required to capture pedigree relationships, is only likely to capture genotyped individuals across one or a few years. As a consequence, environmental variability in space and time is not always accurately captured, which in turn can also lead to inaccuracies in predicting selection response. Finally, heritability models require large sample sizes (e.g., hundreds to thousands of individuals) to produce precise estimates of heritability (de Villemereuil, 2012). While genomic relatedness may be constructed for thousands of individuals, pedigrees provide a cost‐effective approach for sampling IBD across more individuals over time.

Methods that identify the regions of the genome that contribute to trait heritability, broadly termed “gene mapping”, often require or are enhanced by pedigree information. Linkage mapping is one important method of gene mapping, which leverages genetic markers, phenotypic data, and recombination across a multigenerational pedigree to understand the general location of genes controlling traits (Laird & Lange, 2011; Slate et al., 2009). Tracking dense panels of genome‐wide markers over generations also forms the basis of genetic linkage maps, which characterise the recombination landscape and show the position and order of genes throughout the genome. For example, a linkage map was created for collared flycatcher (*Ficedula albicollis*) using deep pedigree data and thousands of genome‐wide SNPs, which has provided an understanding of flycatcher genome architecture in comparison to other species (Kawakami et al., 2014). Beyond providing an understanding of genome evolution, these maps provide useful context to how populations are expected to respond to selection pressures (Stapley et al., 2017). For example, mapping resources and pedigrees developed in California condor are being used to understand the genomic basis of chondrodystrophy, a lethal form of dwarfism in this critically endangered species (Ralls et al., 2000; Romanov et al., 2009). Besides traits that are controlled by single genes of large effect, linkage maps and pedigree data can be utilised for quantitative trait locus linkage mapping, or QTL mapping, which enables the detection of many genomic loci that contribute to continuous trait differences (Slate, 2005). For example, QTL mapping identified candidate adaptive loci contributing to bud phenology in white spruce (*Picea glauca*; Pelgas et al., 2011) and phenotypic differences between marine and freshwater nine‐spined stickleback (*Pungitius pungitius*; Yang et al., 2016). Similarly, pedigree information can be incorporated into GWAS, which leverages dense markers, putatively unrelated individuals, and phenotypic information to understand the genomic basis of traits. For example, pedigree data can be combined with GWAS to partition direct and indirect genetic effects on phenotypes of interest, as opposed the GWAS alone which combines these effects (Young et al., 2019). In doing so, a pedigree‐informed GWAS provides an unbiased way to study genetic effects and response to selection. Studies have also shown that GWAS that incorporate pedigree data are better able to avoid type I error and add greater precision to GWAS analyses, especially in data sets with low marker density (Chen et al., 2013; Zhou et al., 2017).

### 2.4 Pedigrees are a bridge between researchers, practitioners, and Indigenous Peoples and Local Communities

Pedigrees are a useful tool for understanding genetics and informing management efforts for threatened species. Beyond these uses, we contend that pedigrees help bridge the gap between conservation research and practice (i.e., the “research‐implementation gap” or the “conservation genomics gap”; Knight et al., 2008; Shafer et al., 2015). Collating and refining pedigree data is a time consuming task that often requires strong communication between practitioners who collect long‐term demographic data sets and researchers who help validate pedigrees and perform downstream analyses. Indeed, the act of building a pedigree requires mutual knowledge of species life history, genealogy, and the genetic data used for validation. This codevelopment of pedigree resources builds trust, which can translate into improved application of genetic and genomic research into the conservation management of threatened species (Box 1).

BOX 1 **Perspectives from conservation practitioners on the benefits of pedigree use in species management. Viewpoints provided by Liz Brown (LB), Daryl Eason (DE), and Anne Richardson (AR)**

We (LB, DE, AR) are conservation practitioners in Aotearoa New Zealand working with three critically endangered endemic bird species with pedigree data: kākāpō (*Strigops habroptilus*; Box 1A), kākāriki karaka (*Cyanoramphus malherbi*; Box1B), and kakī (*Himantopus novaezelandiae*; Box1C). Over the past 5 years, we have collaborated with conservation researchers to integrate pedigrees from long‐term records into digital studbook databases (kākāpō, kakī), validate pedigrees with genetic and genomic data (all), and inform genetic and genomic studies with our research collaborators using pedigree data (all).

Collecting, validating, and managing pedigree data is time intensive. For example, building a pedigree for kakī from scratch and validating it using genetic and genomic data took over 500 h to accomplish (Galla, 2019). However, in our collective experience, creating pedigree resources for these species has been worth the effort. Pedigrees have allowed us to make informed translocation recommendations in kākāpō, pairing recommendations in kakī and kākāriki karaka, and understand founder representation and demographics in all three species. Because kakī is semi‐wild and kākāpō is a wild lek breeder, genetic and genomic data has also been helpful in validating the parentage we have assinged for each offspring. Given our relationships with conservation researchers at universities and research institutes, this data has contributed towards genomic studies for each of these species, including the efforts of Kākāpō 125+, a project to sequence the genomes of all living kākāpō.

The act of collaborating with conservation researchers on building pedigrees has required substantial communication between researchers and practitioners. The time and energy required to communicate (e.g., emails, phone calls, face‐to‐face meetings, workshops) has not only strengthened our pedigree tools and our confidence using them, but has strengthened our relationships. The pedigree acts as a common ground between conservation genetic research and practice, and has contributed towards trust that allows genetic and genomic research to inform management efforts for these critically endangered species. These trusted relationships have built a bridge for us to learn about aligned conservation projects in Aotearoa New Zealand and abroad, and incorporate new approaches for pedigree and species management in our own programs.

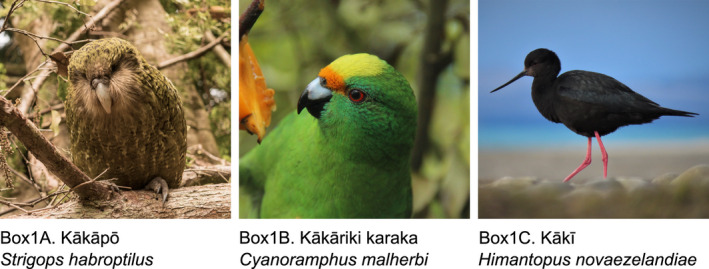

In illustrating connections that link the present to the past, pedigrees are well‐aligned with both Indigenous and non‐Indigenous worldviews (Collier‐Robinson et al., 2019; Hudson et al., 2007, 2020). Given this alignment, pedigrees can provide a centrepoint for discussions with Indigenous decision makers regarding conservation genetics research of culturally significant species (Box 2). Further, in our experience working with Indigenous Peoples and Local Communities (IPLC) to enhance the recovery of threatened taonga (treasured) species in Aotearoa New Zealand, discussing familial ties between individuals provides opportunities for all parties to share diverse knowledge regarding these individuals as well as the environments around them. Given this, we encourage researchers to consider the use of pedigrees to help build mutually beneficial relationships with IPLC.

BOX 2 **Pedigrees for culturally significant species help grow relationships with Indigenous Peoples and Local Communities. Viewpoints provided by Yvette Couch‐Lewis (YCL), Stephanie J. Galla (SJG) and Tammy E. Steeves (TES)**

We are conservationists representing mana whenua (those with local tribal or sub‐tribal authority, YCL) and genetic researchers (SJG, TES) who work in partnership to enhance the recovery of threatened species treasured by Māori (the Indigenous Peoples of Aotearoa New Zealand).

For Māori, whakapapa is a genealogical framework that describes the origins and relationships of all things, linking plants, animals, and people with the environment including the mountains, rivers, and winds, across time and space (Roberts, 2013; Tau, 2001). Ultimately, it is whakapapa that connects individuals to each other, to their ancestors, and to the land (Tau, 2001; Te Rito, 2007). Given this worldview, it is no wonder that relationships between researchers and mana whenua are enriched by pedigrees, which provide a visualization of whakapapa.

YCL is kaitiaki (guardian) for kākāriki karaka, or orange‐fronted parakeet, on behalf of Ngāi Tahu, a large tribe on the South Island of Aotearoa New Zealand. When deciding which individual to choose for the kākāriki karaka reference genome, SJG and TES provided a pedigree showing the whakapapa of all individuals available for sampling. Using this resource, YCL chose Maverick, a captive male bird. From the pedigree, she learned that his ancestors included individuals from the last three remnant populationson the mainland (Box 2A) and that he was a good father (he had many offspring; data not shown). Simply put, Maverick provided a link between the past—and the future—of kākāriki karaka with the land, and his genome remains an invaluable resource for the conservation genomic management of this critically endangered bird from Aotearoa New Zealand (e.g., Galla et al., 2020).

Beyond kākāriki karaka, based on our collective experience working with other threatened taonga (treasured) species in Aotearoa New Zealand, we are confident pedigrees will continue to help grow our relationships with mana whenua—as well as local communities—leading to improved conservation outcomes. We encourage those interested in building trusted relationships with Indigenous Peoples and Local Communities to seek guidance relevant to their local context and to engage in the extensive scholarship already available (e.g., Chambers et al., 2021; Collier‐Robinson et al., 2019; Polfus et al., 2016).

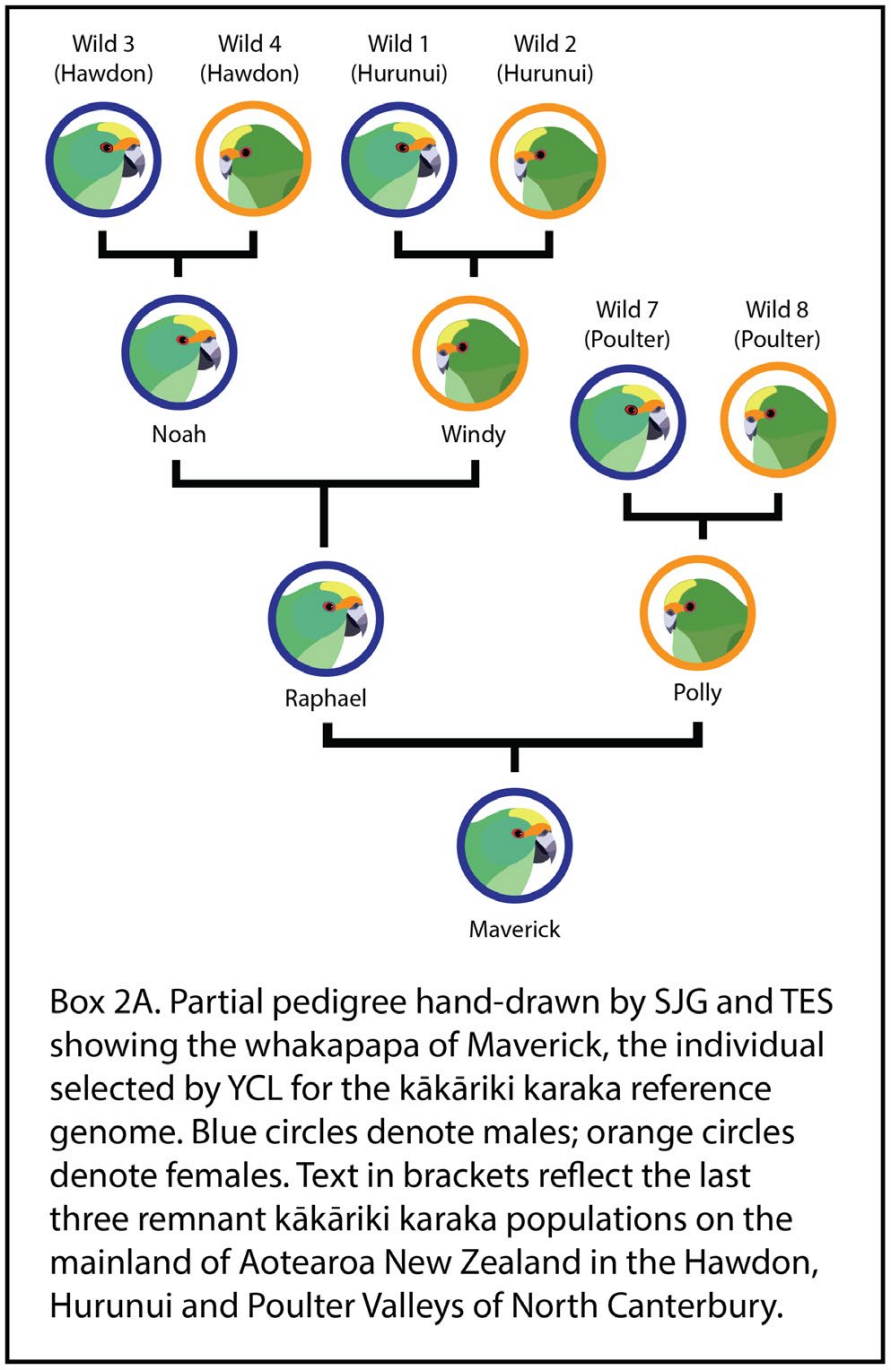

We have also found that pedigrees provide a helpful visual to communicate fundamental conservation genetics concepts like relatedness, inbreeding, and heritability without jargon to non‐scientific audiences, in large part because pedigrees are relatable. Indeed, our collective coauthorship has had experience using pedigrees–generally presented as family trees–as highly effective tools for engaging with school groups, university classrooms, retirees, policy makers, and politicians regarding conservation genetic management of threatened species. Effective science communication enhances conservation outcomes (see Holderegger et al., 2019), and we are confident that pedigrees will remain an important tool for science communicators for years to come.

## 3 A CONSERVATION GENOMIC POWERHOUSE

While pedigrees are one of the most long‐standing tools in the conservation genetics toolbox, when coupled with unprecedented advances in genetics and genomics, they create a powerhouse for conservation management capable of better characterizing and preserving diversity in a changing world. Robust pedigrees —that can be complemented and completed with molecular data— continue to provide long‐term demographic and life history information that allow for improved research design and implementation and provide opportunities to build and maintain trusted relationships. We contend that pedigrees will remain relevant for applied and fundamental research and advocate for their maintenance in programs across the ex situ and in situ management spectrum.

For those beginning to assemble pedigree data, we offer the following advice for maximized success. First, when establishing a new pedigree of plants or animals, collect tissue samples and metadata (e.g., phenotypic information and geographic provenance) for as many individuals as possible (including all founders, in the case of conservation breeding programs) to ensure you can inform inter‐individual relationships. In addition to initial sampling, we recommend maintaining detailed metadata (e.g., date of birth, sex, morphometrics of interest, cause of death) and tissue samples for all pedigreed individuals in subsequent generations. Tissue banks for all pedigreed individuals can help jumpstart genomic research, coupled with collected metadata embedded in the pedigree. Tissue samples can also be used by conservation genetic researchers to periodically validate the pedigree for accuracy and assist with any uncertain parentage assignments. Finally, we advocate for clear communication between researchers, practitioners, and IPLC to ensure pedigrees continue to be built and used as a tool for management for generations to come.

## AUTHOR CONTRIBUTIONS

The conception of this piece was led by Stephanie J. Galla and Tammy E. Steeves, with support from all authors. Writing and illustrations were led by Stephanie J. Galla with contributions from all authors. Box 1 was led by Liz Brown, Daryl Eason, and Anne Richardson, with support from Stephanie J. Galla. Box 2 was written by Stephanie J. Galla, Yvette Couch‐Lewis (Ngāi Tahu: Te Hapū o Ngāti Wheke, Ngāti Waewae), and Tammy E. Steeves.
